# 1844. HIV Home Care - The First Five Years

**DOI:** 10.1093/ofid/ofad500.1672

**Published:** 2023-11-27

**Authors:** Sumaiya F Khaja, Vishakh C Keri, Tammie McClendon, Jennifer Veltman, Gretchen Snoeyenbos Newman

**Affiliations:** Wayne State University School of Medicine, Rochester Hills, Michigan; Wayne State University, Detroit, Michigan; Wayne State University, Detroit, Michigan; Loma Linda University School of Medicine, Linda Loma, California; Wayne State University School of Medicine, Rochester Hills, Michigan

## Abstract

**Background:**

To achieve HIV care continuum goals, novel modes of HIV care are needed. In 2017, the Wayne State University Home Care Program (HCP) was launched in Detroit, Michigan. The primary purpose of this program is to re-engage people with HIV (PWH) who have been unable to attend regular clinic visits through an intensive, in-home model of HIV care. This study evaluates the outcomes of the first 5 years of this program.

**Methods:**

We conducted a retrospective cohort analysis of the 81 patients enrolled in the HCP from inception in 2017 to December, 2022. We evaluated duration of participation, reason for discharge from the program, and effect of a full year of program participation on viral suppression (viral load ≤ 20 copies/mL) and CD4 count (above or below 200 cells/mm^3^). Patients who did not have at least 1 year of participation were excluded from analysis of change in VL and CD4 count. McNemar test of significance was used, p value of < 0.05 was considered significant.

**Results:**

Most of the patients enrolled in the HCP were African American (95.1%) and male (65.4%). Common primary barriers to clinic visits were transportation (27.2%) and stigma (25.9%). The median duration spent in the HCP was 924 days (approximately 2.5 years). At entry, 15.3% of patients had a suppressed viral load, and after the first year of enrollment, 52.5% of patients achieved viral load suppression (p< 0.001). Similarly, 72.9% of patients achieved a CD4 ≥ 200 cells/mm^3^ after a year when compared to 57.6% of patients at entry with CD4 ≥ 200 cells/mm^3^ (p=0.012). Of the 81 patients enrolled during the study period, 28 patients (50%) returned to clinic-based care, out of which 21 patients (91.3%) were retained in care and 2 (8.7%) were lost to follow-up. Total of 11 patients (19.6%) died during the study period.

**Figure:**
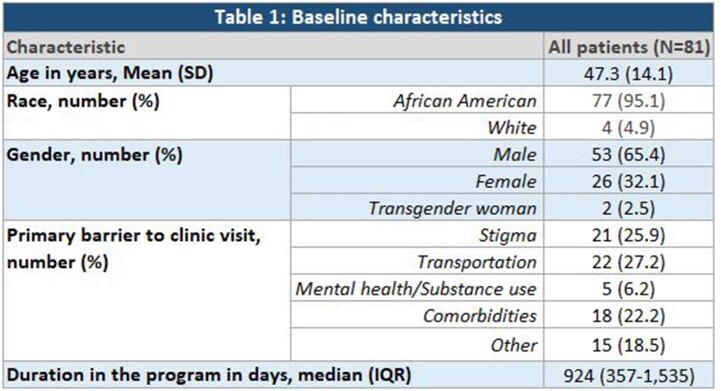

**Table 2:**
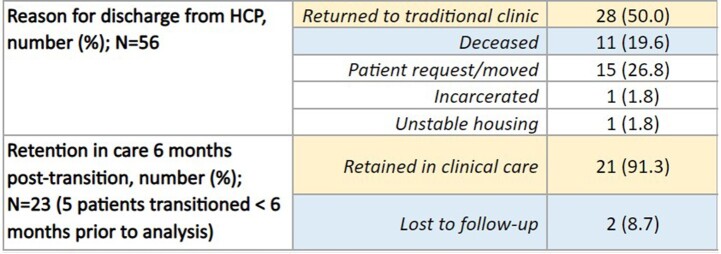
Home Care Program Outcomes

**Figure:**
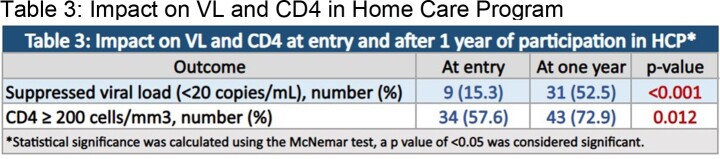

**Conclusion:**

The Wayne State University Home Care Program is an innovative model to re-engage and maintain PWH who are unable to receive care in traditional settings. By removing physical and stigma-related barriers, the HCP was able to improve viral load suppression and CD4 count among an ill and “hard to reach” cohort. Additionally, the HCP empowered patients to successfully return to clinic-based care.

**Disclosures:**

**All Authors**: No reported disclosures

